# Stable DNA Sequence Over Close-Ending and Pairing Sequences Constraint

**DOI:** 10.3389/fgene.2021.644484

**Published:** 2021-05-17

**Authors:** Xue Li, Ziqi Wei, Bin Wang, Tao Song

**Affiliations:** ^1^The Key Laboratory of Advanced Design and Intelligent Computing, Ministry of Education, School of Software Engineering, Dalian University, Dalian, China; ^2^School of Software, Tsinghua University, Beijing, China; ^3^College of Computer and Communication Engineering, China University of Petroleum, Qingdao, China

**Keywords:** DNA computing, DNA sequence design, constraint, WOA, ICW

## Abstract

DNA computing is a new method based on molecular biotechnology to solve complex problems. The design of DNA sequences is a multi-objective optimization problem in DNA computing, whose objective is to obtain optimized sequences that satisfy multiple constraints to improve the quality of the sequences. However, the previous optimized DNA sequences reacted with each other, which reduced the number of DNA sequences that could be used for molecular hybridization in the solution and thus reduced the accuracy of DNA computing. In addition, a DNA sequence and its complement follow the principle of complementary pairing, and the sequence of base GC at both ends is more stable. To optimize the above problems, the constraints of Pairing Sequences Constraint (PSC) and Close-ending along with the Improved Chaos Whale (ICW) optimization algorithm were proposed to construct a DNA sequence set that satisfies the combination of constraints. The ICW optimization algorithm is added to a new predator–prey strategy and sine and cosine functions under the action of chaos. Compared with other algorithms, among the 23 benchmark functions, the new algorithm obtained the minimum value for one-third of the functions and two-thirds of the current minimum value. The DNA sequences satisfying the constraint combination obtained the minimum of fitness values and had stable and usable structures.

## Introduction

DNA computing is a new and promising interdisciplinary subject based on computational science and molecular biology, which shows great potential in solving NP problems (Wang et al., 2019; Zhu et al., 2020). At the end of the 20th century, Adleman (1994) used DNA molecules for calculation and solved the Hamiltonian problem (Heidari, 2014). The successful solution of this problem led DNA computing to become a field of great development. It has since been widely used to solve problems in many domains, including PCR amplification (Sze and Schloss, 2019), DNA sequencing (Shendure et al., 2017), bioinformatics (Zou and Liu, 2019), prediction of disease genes (Zeng et al., 2020a,b), image encryption (Zhou et al., 2020), and DNA data storage (Zhang et al., 2019; Cao et al., 2021), among others.

Benenson et al. (2004) made decisions through simple Boolean logic and successfully used RNA interference to construct molecular computing cores in human kidney cells. Yaakov et al. (Rinaudo et al., 2007) announced a breakthrough DNA computer, which can theoretically release anticancer drugs into cancer cells. In 2017, researchers used the CRISPR-Cas system (Shipman et al., 2017) to encode the pixel values of black-and-white images and short films into the genome of living bacterial populations; they minimized the technical limitations of the information storage system. Thubagere et al. (2017) developed a DNA robot that can control DNA to perform specific actions, such as picking and sorting goods in solution. Han et al. (2017) developed a new strategy called single-stranded origami (ssOrigami), which uses a single-stranded DNA or RNA as long as thin as a noodle to implement a self-folding structure without a topological junction, which could allow drugs to travel directly to the site of injury within the cell. Li et al. (2018) developed a nanorobot based on DNA origami technology that can be used to carry thrombin to accurately target tumor cells, and more broadly, this technology can be used for many types of cancer. Cherry and Qian (2018) used the extended seesaw motif DNA neural network for pattern discrimination and constructed a neural network using DNA sequence to realize the recognition of handwritten digits in model organisms. Palluk et al. (2018) proposed a strategy for the synthesis of oligonucleotides using a template-independent polymerase terminal deoxyribonucleotide transferase and obtained a scheme to repeatedly write a definite sequence. Schickinger et al. (2018) proposed DNA origami for creating a tethered multifluorophore movement experiment and explain interactions between cells. This is easy to use and has a wide range of applications. In order to avoid errors in DNA storage, Deng et al. (2019) adapted a hybrid coding system, which is composed of improved variable length run-length limited (vl-rll) codes and optimized photograph low-density parity check codes (LDPCs). Wang et al. (2020) proposed a deep learning framework, SeqEnhDL, to classify cell type–specific enhancers based on sequence features. This framework can transform folding changes of any DNA sequence into deep learning model features. Zhang et al. (2020) constructed a DNA molecular lock by using the characteristics of enzyme mutual inhibition and realized the information protection function at the molecular level.

In addition, in the face of massive data, the current computer is limited in terms of data storage and computing speed. Biomolecular computers have attracted the interest of scientists. DNA computer, as one of the biomolecular computers, has received much attention due to its small size, large storage capacity, fast operation, low energy consumption, and high parallelism. DNA computers use DNA (deoxyribonucleic acid) as a basis to bind enzymes for biochemical reactions that eventually generate DNA sequences carrying specific genetic information. These sequences are used to perform computation and solve the problem. DNA computing is encoded by A, T, C, and G, which is different from the binary combination of the traditional computer.

The design of DNA sequences is the key to perform DNA computing, and the quantity and quality of sequences can directly affect the accuracy and efficiency of calculations. Therefore, a good coding method is of great significance to improve the reliability and accuracy of DNA computing. Deb et al. (2002) proposed a fast, non-dominated sorting genetic algorithm, which was used to solve a class of multi-objective optimization problems. With the help of linear coding, Gaborit and King (2005) developed a DNA code that met the anti-complement constraint and GC content requirement. Thus, they constructed an appropriate DNA sequence set. Shin et al. (2005) carried out multi-objective optimization for DNA sequences, including continuity, similarity, hairpin structure, H-measure, and GC content. Because the traditional algorithm cannot address the heterogeneity and conflict of DNA sequences, scientists designed a multi-objective optimization algorithm based on the artificial bee colony (MO-ABC) (Chaves-González et al., 2013), in which six kinds of conflict problems were solved, and finally, a reliable DNA sequence was generated. In 2014, in order to obtain effective DNA sequences, they proposed to use the multi-objective differential evolution algorithm (DEPT) (Chaves-González and Vega-Rodríguez, 2014) to optimize DNA sequences. In 2015, they used a hybrid multi-objective heuristic algorithm (H-MO-TLBO) (Chaves-González, 2015) to design DNA sequences. Yang et al. (2017) proposed to add the niche exclusion mechanism to improve the invasive weed optimization algorithm, which enhanced robustness and obtained the optimal sequence. Wang et al. (2018) improved the fast non-dominated sorting genetic algorithm II (INSGA-II) and achieved a high convergence rate and reliable DNA sequences. In 2019, in order to further optimize the DNA sequence, Chaves-González and Martínez-Gil (2019) introduced an algorithm called pMO-ABC that harvested different numbers of DNA sequences. In 2020, to decrease the error rate, Cao et al. (2020) presented a new constraint, namely, uncorrelated address, and constructed a set of effective DNA codes. Yin et al. (2020) considered multiple constraints to ensure accurate hybridization of DNA sequences.

In this study, to obtain a high-quality DNA sequence set, the constraints of Close-ending and Pairing Sequences Constraint (PSC) were added to the original constraint combination to form a new combinatorial constraint. The PSC addresses non-specific hybridization that occurs within the DNA sequences set, and the constraint adds a sliding method to ensure that each base can be traversed. Adding PSC to the DNA sequence set reduces the probability of interaction between sequences. The reason for the Close-ending constraint is that the G base and the C base in the sequence have three hydrogen bonds, and there are only two hydrogen bonds between the A base and the T base, so the stability of the AT end of the sequence is less than that of the G-C end. The DNA sequence reacts according to the principle of base complementary pairing. When G-C base is selected as the terminal of the sequence, the desired structure can be achieved when the DNA sequence continues to react. In addition, the constraint also include continuity, hairpin structure, H-measure, similarity, GC content, melting temperature, triplet-bases unpaired. The first four constraints are used as objective functions to calculate the fitness value; the remaining constraints are used to narrow the solution space. At the same time, we enrich and improve the WOA algorithm. In addition, the predatory behavior of another marine mammal, manta ray (Zhao et al., 2020), is added to expand the predation range and maintain the diversity of the population. To improve the global search ability, the sine cosine model (Mirjalili, 2016) is combined with chaos (Shan et al., 2005) to further expand the solution space. After 23 benchmark functions, it is proved that the Improved Chaos Whale (ICW) optimization algorithm is meaningful. It reached the optimal value in most test functions. Under the combined action of the new constraint combination and the improved algorithm, excellent DNA sequences can be selected as elites. These elite sequences have a minimum value of zero in continuity and hairpin structure and the current minimum value in H-measure, followed by the minimum melting temperature change. In the evaluation of NUPACK, the concentrations of all DNA sequences before and after entering the solution were normalized to total values. All sequences showed stable and usable structures, indicating that the DNA sequences have good stability.

The rest of this article is arranged as follows. The second part introduces the constraints of constructing the DNA sequence set, including the new Close-ending constraint and PSC. The third part introduces the ICW optimization algorithm. In the fourth part, the results of fitness analysis and NUPACK evaluation are given. The last part is the summary and conclusion.

## The Constraints on DNA Sequence Design

To ensure the accuracy of DNA calculation and avoid non-specific hybridization of sequences, constraints must be imposed on DNA sequences. The construction of useful and high-quality DNA sequence set is dependent on strict constraints, which can enhance the robustness of the sequences. Continuity and hairpin structure constraints can effectively prevent sequences from generating secondary structures. The addition of triplet-bases unpaired and PSC to the sequences without secondary structure can not only avoid the self-complementary reaction but also effectively avoid the reaction between the sequences to generate other structures. On this basis, by adding similarity and H-measure, well-structured sequences be obtained that reduce unnecessary hybridization with other sequences. The addition of GC content and melting temperature constraints can keep the sequences in a thermodynamically stable state. Combined with Close-ending constraint, the formed DNA double strand is also stable in structure. Applying these constraints can lead to good sequences. In this study, we adopted all the above constraints in the design sequences.

### Continuity

Continuity (Chaves-González et al., 2013) refers to the fact that the same bases are displayed side by side in a confined area. The continuous presence of the same base in a limited region can cause the DNA sequence to stack or distort. To avoid such a secondary structure of the DNA sequence, it is necessary to select a DNA sequence with little continuity. The continuity can be visualized with the following example. Assuming that the DNA sequence threshold is 4, then in the sequence TTAGGGATCCATTTTT, the last sub-sequence with an underscore will trigger the threshold. To improve the quality of DNA sequences, such sequences will be removed from the sequence set. The mathematical formula is as follows:

(1) $$f_{c\,o\,n}\,\left(L\right)=\sum_{p=1}^{m}C\,o\,n\,\left(L_{p}\right)$$

(2) $$C\,o\,n\,\left(x\right)=\sum_{i=1}^{n-C\,T}T\,\left(c\,o\,n\,t_{a}\,\left(x,i\right),C\,T\right)$$

(3) $$c\,o\,n\,t_{a}\,\left(x,i\right)=\left\{ \begin{array}{cc} c\,i\,f\,\exists c,s.t.x_{i}\neq a,x_{i+j}=a\,f\,o\,r\,1\leq j\leq c & \\ a\,n\,d\,x_{i+c+1}\neq a & \\ 0\,o\,t\,h\,e\,r\,w\,i\,s\,e & \end{array}\right.$$

where *m* is the number of DNA sequence sets; *L*_*p*_ is a sequence in the DNA set *L*; *n* is the number of bases in the current DNA sequence; *CT* is a specific continuity threshold; *T (b, CT)* is a threshold function; when *b* > *CT*, the result is *b*; otherwise, the result is 0. *cont_*a*_ (x, i)* returns the number of consecutive bases, where a ∈{A, T, C, G}.

### Hairpin Structure

Hairpin structure (Chaves-González et al., 2013) is a secondary structure caused by the stacking of the DNA sequence itself, which may lead to inaccurate calculation. The hairpin structure is composed of a hair ring and hair stem. The number of bases for the hairpin to form the smallest ring is *R*_*min*_, and *P*_*min*_ is the minimum length of the hairpin stem. The mathematical formula for calculating the hairpin value is as follows:

(4) $$f_{h\,a\,i\,r\,p\,i\,n}\,\left(L\right)=\sum_{\rho =1}^{m}H\,a\,i\,r\,p\,i\,n\,\left(L_{p}\right)$$

(5) $$H\,a\,i\,r\,p\,i\,n\,\left(x\right)=\sum_{q=P_{m\,i\,n}}^{\left(n-R_{m\,i\,n}\right)}\sum_{r=R_{m\,i\,n}}^{n-2\,q}\sum_{i=1}^{n-2\,q-r}T$$

(6) $$P\,L_{q\,r\,i}=m\,i\,n\,\left(p+i,l-r-i-p\right)$$

where *r* is the ring length of the hairpin structure, and *q* is the stem length. *m* is the number of DNA sequence sets, and *n* is the number of bases in a DNA sequence. For *T (a, y)*, when *a* > *y*, the result is *a*; otherwise, it is 0. The function *cb (a, b)* means that when *a* and *b* are complementary, the result is 1; otherwise, the result is 0. The equations should be inserted in editable format from the equation editor.

### H-Measure

H-measure (Chaves-González, 2015) is a parameter to measure the degree of sequence hybridization. The parameter records the number of complementary bases of two sequences. The calculation formula is as follows:

(7) $$f_{H-m\,e\,a\,s\,u\,r\,e}\,\left(L\right)=\sum_{i=1}^{m}\sum_{j=1,i\neq j}^{m}H-m\,e\,a\,s\,u\,r\,e\,\left(L_{i},L_{j}\right)$$

where *m* represents the size of sequence *L* sets; and *L*_*i*_ and *L*_*j*_ represent two sequences in opposite directions. The H-measure is classified into two types: continuous and discontinuous.

(8) $$H_{m\,e\,a\,s\,u\,r\,e\,\left(x,y\right)}=M\,a\,x_{g,i}\,\left(h_{d\,i\,s}\,\left(x,s\,h\,i\,f\,t\right)\,\left(y\,{\left(-\right)}^{g}\,y,t\right)\right)+h_{c\,o\,n\,t}\left(x,shift\right)\left(y{\left(-\right)}^{g}y,t\right))$$

where *x* and *y* represent different DNA sequences. The shift function defines the offset from *y* to t.

(9) $$h_{d\,i\,s}\,\left(x,y\right)=T\,\left(\sum_{i=1}^{n}b\,p\,\left(x_{i},x_{j}\right),H_{d\,i\,s}\times l\,e\,n\,g\,t\,h_{n\,b}\,\left(y\right)\right)$$

(10) $$h_{c\,o\,n\,t}\left(x,y\right)=\sum_{i=1}^{n}T(cbp\left(x,y,i\right),H_{c\,o\,n\,t}$$

where *H*_*dis*_ is a number between 0 and 1; *H*_*con*_ is a positive integer from 1 to *n*; and the function *cbp (x, y, i)* represents the length of a continuous base pair starting from the *i*th base of the sequence.

(11) $$b\,p\,\left(x,y\right)=\left\{ \begin{array}{cc} 1\,x=y & \\ 0\,o\,t\,h\,e\,r\,w\,i\,s\,e & \end{array}\right.$$

(12) $$c\,b\,p\,\left(x,y,i\right)=\left\{ \begin{array}{cc} c\,i\,f\,\exists c,s.t.b\,p\,\left(x_{i},y_{j}\right)=0\,b\,p\,\left(x_{i+j},y_{i+j}\right)=1 & \\ f\,o\,r\,1\leq j\leq c\,a\,n\,d\,b\,p\,\left(x_{i+c+1},y_{i+c+1}\right)=0 & \\ 0\,o\,t\,h\,e\,r\,w\,i\,s\,e & \end{array}\right.$$

### Similarity

Similarity (Chaves-González, 2015) is an important index to evaluate sequence diversity. The higher the similarity, the more likely it is that non-specific hybridization will occur. It can calculate the number of the same base after the shift of two identical sequences. The higher the number of the same base, the more similar is the coding. Similarity is divided into discontinuous similarity and the largest continuous common subset. The formula for calculating similarity is as follows:

(13) $$f_{s\,i\,m}\left(L\right)=\sum_{i=1}^{n}\sum_{j=1}^{n}Max_{g,t}(s_{d\,i\,s}\left(x,Shift\left(y{\left(-\right)}^{g}y,t\right)\right)+s_{c\,o\,n\,t}\left(x,Shift\left(y{\left(-\right)}^{g}y,t\right)\right))$$

where *L* is the set of DNA sequences; *n* is the number of set *L*; and *x* and *y* are the different sequences in set *L*. (-) indicates a gap. Shift represents the offset of the encoding *y* through *t*. *g* ∈ [0, 3].

(14) $$s_{d\,i\,s}\,\left(x,y\right)=T\,\left(\sum_{i=1}^{n}e\,q\,\left(x_{i},y_{i}\right),D\,S\times n\right)$$

(15) $$s_{c\,o\,n\,t}\,\left(x,y\right)=\sum_{i=1}^{n}T\,\left(c\,e\,q\,\left(x,y,i\right),C\,S\right)$$

(16) $$e\,q\,\left(x,y\right)=\left\{ \begin{array}{cc} 1\,x=y & \\ 0\,o\,t\,h\,e\,r\,w\,i\,s\,e & \end{array}\right.$$

(17) $$c\,e\,q\,\left(x,y,i\right)=\left\{ \begin{array}{cc} c\,i\,f\,\exists c,s.t.e\,q\,\left(x_{i},y_{j}\right)=0\,e\,q\,\left(x_{i+j},y_{i+j}\right)=1 & \\ f\,o\,r\,1\leq j\leq c\,a\,n\,d\,e\,q\,\left(x_{i+c+1},y_{i+c+1}\right)=0 & \\ 0\,o\,t\,h\,e\,r\,w\,i\,s\,e & \end{array}\right.$$

For *T (a, value)*, when *a* > *value*, the result is *a*; otherwise, it is 0. *ceq (x, y, i)* is the length of the continuous common subsequence starting from the ith base of the sequence. DS is a real number from 0 to 1; CS is a positive integer from 1 to *n*.

### Melting Temperature

The melting temperature (Yang et al., 2017) of DNA is an important parameter. In the process of DNA denaturation, double stranded DNA molecules undergo physical changes. In the process of denaturation from double strand to single strand, the temperature at which half of the DNA molecules are released is called the melting temperature. This behavior is an important constraint to ensure the thermodynamic stability of DNA molecules. The melting temperature is usually calculated by the gas chromatography content method and the nearest neighbor method. In this article, the melting temperature is calculated by the nearest neighbor method. The calculation formula is as follows:

(18) $$T_{m}=\Delta \,H^{\circ }/\left(\Delta \,S^{\circ }+R\,\ln C_{t}\right)$$

where Δ*H* and Δ*S* represent the standard enthalpy change and entropy change in the hybridization reaction, respectively, and the calculation method is the same as that of the free energy change. *C*_*t*_ is the molar concentration of DNA molecules. When the molecule is a symmetric sequence, its molar concentration is *C_*t*_/4*. *R* is the gas constant of 1.987 cal/Kmol.

### GC Content

GC content (Yang et al., 2017) is the ratio of guanine and cytosine in a DNA sequence. GC content is an important constraint; it can directly affect the stability of a DNA sequence. In a DNA sequence, the number of bases is expressed by *n*; the number of guanine is *a*; and the number of cytosine is *b*. Generally speaking, the GC content (*t*) of a sequence is

(19) $$t=\frac{a+b}{n}*100\%$$

Then, in the sequence GTTCGTACTGATCGTAGC, the GC content is (5+4) /18^∗^100%; that is, 50%.

### Triplet-Bases Unpaired

Triplet-bases unpaired (Li et al., 2020) is employed to avoid the complementary reaction of DNA sequence in solution. The sequence entered into NUPACK is evaluated, and the result is denoted by *i*. *i = C_*output*_/C_*input*_*; *C*_*output*_ is the sum of the sequence concentrations in the solution after NUPACK input; and *C*_*input*_ is the sum of the DNA sequence concentrations when NUPACK is input. The closer *i* is to 1, the higher is the sequence quality. X is a DNA sequence; n is the base number of X; Y is the inverse sequence of X; and *x*, *y* are the subsequences of X and Y. It is expressed by the following formula:

(20) $$f_{p\,a\,i\,r}\,\left(x\right)=\left\{ \begin{array}{cc} p\,a\,i\,r\,\left(x\right) & s\,u\,b\,c\,b\,\left(x,y,k\right)=3\\ x & s\,u\,b\,c\,b\,\left(x,y,k\right)\neq 3 \end{array}\right.$$

where *x = (x_*i*_, x_*i*__+__1_, x_*i*__+__2_)*; *y = (y_*j*_, y_*j*__+__1_, y_*j*__+__2_)*; and *i, j∈[1, n−2]*. The function *subcb(x, y, k)* calculates the number of base complementary pairs starting from the *k*th base.

### Close-Ending

**FIGURE 1:**
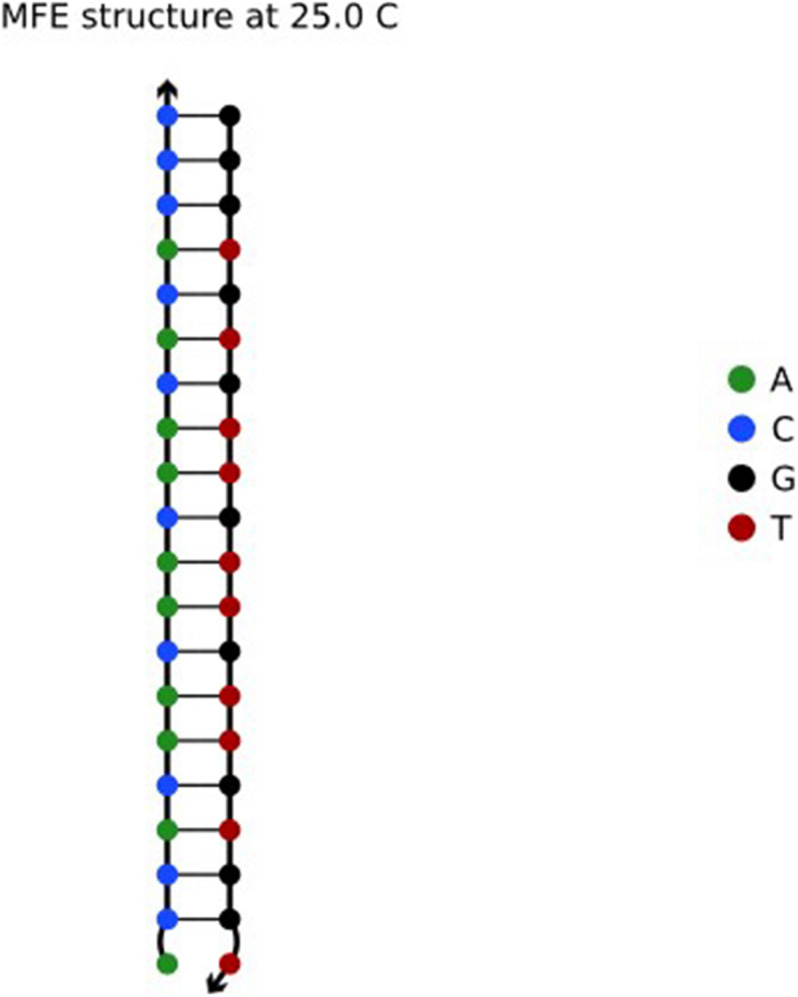
Sequence ACCACAACAACAACACACCC and its complementary sequence.

In a pair of complementary DNA sequences, the sequence usually creates a gap at one end of the A-T base pair, resulting in an unstable structure (Chaves-González and Vega-Rodríguez, 2014) of the sequence in solution. There are three hydrogen bonds between the G and C bases in the DNA sequence, but there are only two hydrogen bonds between A and T, so the bond strength formed by A-T is less than that between G-C. The structure evaluated in NUPACK is shown in Figure 1.

As shown in Figure 1, one end of the sequence is G-C. Here, the reaction takes place according to the principle of complementary base pairs. However, there is a gap at one end of the A base and T base, which is less stable than that of GC (Zgarbova et al., 2014). Therefore, by choosing GC base as the port of the sequence, the DNA sequence in the solution can achieve the desired structure. Based on the above, the Close-ending constraint is proposed. Assuming that *X* is a DNA sequence and *X*_*i*_ is the ith base in the DNA sequence, then

(21) $$X=x_{1}\,x_{2}\,\ldots \,x_{i}\,\left(x_{1},x_{i}\,\epsilon \,\left\{G,C\right\}\right)$$

### PSC

**FIGURE 2:**
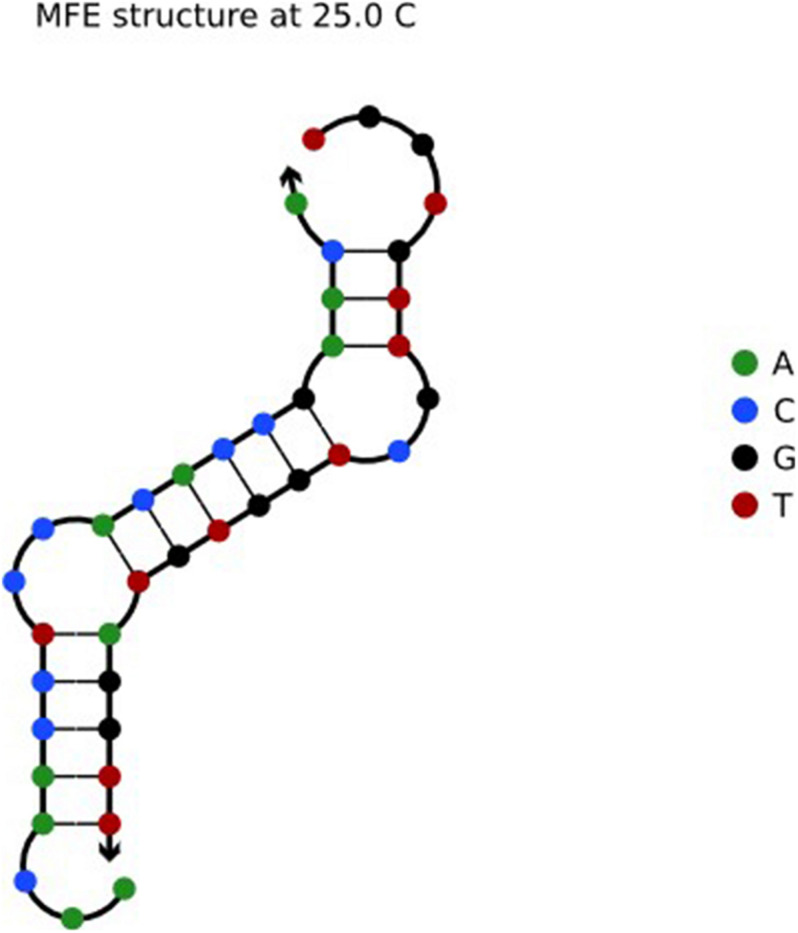
Sequence a: AACAACCTCCACACCGAACA and b: TGGTGTTGCTGGTGTAGGTT.

In solution, DNA reacts in accordance with the principle of base pairing. When the optimized sequences are put into the solution, there will be a reaction between different sequences. Seven optimized sequences in the article (Chaves-González and Martínez-Gil, 2019) were put into NUPACK for evaluation. The sequence a:AACAACCTCCACACCGAACA reacted with sequence b:TGGTGTTGCTGGTGTAGGTT, and *i*(*ab*) = 0.57/2 = 0.285. The structural results are shown in Figure 2.

As shown in Figure 2, sequences *a* and *b* reacted in solution to form another structure. *i* = 0.285 means that the sequences react with each other in the solution. In DNA computing, if a DNA sequence in the solution reacts because of the complementary base pairs, the proportion of the DNA sequence in the solution will be reduced, which affects the accuracy of DNA computing. To solve for the reaction between DNA sequences in solution, the PSC is proposed. Different from the Hamming distance constraint, this constraint adds a sliding method to ensure that every base in the sequence can be traversed. Compared with the H-measure, the comparison between the original sequence and inverse sequence has lower complexity, and the method is simple. Take the a and b sequences as an example. The H-measure calculated values are all 17, while the PSC calculated values are 0. The sequence set, which is constrained by the PSC, reduces the probability of interaction between sequences. This constraint is expressed by the formula:

(22) $$f\,\left(L\right)=\sum_{i=1}^{n}\sum_{j=i+1}^{n}I\,n\,d\,e\,p\,\left(L_{i},L_{j}^{\prime }\right)$$

where *n* is the number of DNA sequences in the *L* set; *L*_*i*_ is the *i*th sequence; *L*_*j*_ is the *j*th sequence; and *L*_*j*_′ is the inverse sequence of *L*_*j*_. *x* and *y* represent two different sequences, and the function *cbp (x, y, i)* represents the length of a continuous base pair starting from the *i*th base of the sequence, where *x*′ represents the sequence of five consecutive bases in the *x* sequence and *y*′ represents the sequence of five consecutive bases in the y sequence. The value of M is four. The value of M is explained in the Supplementary Material.

(23) $$I\,n\,d\,e\,p_{\left(x,y\right)}=\left\{ \begin{array}{cc} x,d=0 & \\ 0,d>M & \end{array}\right.$$

(24) $$d\,\left(x,y\right)=\sum_{a=1}^{m-4}\sum_{b=1}^{m-4}T\,\left(c\,b\,p\,\left(x_{a}^{\prime },y_{b}^{\prime },i\right),M\right)$$

## Algorithm

### The Whale Optimization Algorithm and Chaos Map

The whale optimization algorithm (WOA) (Mirjalili and Lewis, 2016), a kind of meta starting algorithm, is an effective swarm intelligence optimization algorithm. Compared with other group optimization algorithms, the WOA algorithm has the advantages of simple structure and less adjustment parameters. The predation method is to select a random or the current optimal whale position to simulate the behavior of whale predation. The main inspiration of the whale algorithm design is from the unique whale predation method: bubble net predation. In 2016, Mirjalili et al. simulated the predatory behavior of whales with the contraction closed mechanism and spiral update position and selected the random number *p* as the boundary of the two behaviors. To ensure the scientificity and fairness of the data, 0.5 was taken as the threshold value.

When *p* < 0.5, the whales use the contraction closure mechanism to prey. In particular, we need to determine the absolute value of *A* in relation to 1. If the absolute value of *A* is less than 1, select the current optimal whale position to simulate whale hunting behavior; otherwise, the random position of the whale is selected to simulate the predatory behavior of the whale. It is expressed as follows:

(25) $$\vec{A}=2\,\vec{a}\cdot \vec{r}-\vec{a}$$

(26) $$\vec{X}\,\left(t+1\right)=\left\{ \begin{array}{cc} {\vec{X}}^{*}\,\left(t\right)-\vec{A}\cdot \vec{D} & \left|A\right|<1\\ \vec{X_{r\,a\,n\,d}}-\vec{A}\cdot \vec{D} & \left|A\right|\geq 1 \end{array}\right.$$

and

(27) $$\vec{C}=2\cdot \vec{r}$$

(28) $$\vec{D}=\left\{ \begin{array}{cc} \left|\vec{C}\cdot \vec{X^{*}}\,\left(t\right)-\vec{X}\,\left(t\right)\right|\,\left|A\right|<1 & \\ \left|\vec{C}\,\vec{X_{r\,a\,n\,d}}-\vec{X}\right|\,\left|A\right|\geq 1 & \end{array}\right.$$

where $\vec{a}$ decreases from 2 to 0 as the number of iterations increases; and $\vec{r}$ is a random number from 0 to 1.

When *p* ≥ 0.5, to further expand the scope of whale predation, the whale algorithm is improved by adding a new predator–prey method. The added predator–prey mechanism is somersault foraging by learning manta ray simulation. The general predation formula is as follows:

(29) $$\vec{X}\,\left(t+1\right)=\left\{ \begin{array}{cc} \vec{D^{\prime }}\cdot e^{l}\,\cos\left(2\,\pi \,l\right)+\vec{X^{*}}\,\left(t\right) & c<0.5\\ \vec{X}\,\left(t\right)+S\cdot \left(r_{1}\cdot \vec{X^{*}}\,\left(t\right)-r_{2}\cdot \vec{X}\,\left(t\right)\right) & c\geq 0.5 \end{array}\right.$$

where $\vec{D^{\prime }}$ represents the distance between the current whale and the optimal whale; *l*∈[*−*1,1]; the default value of *S* is 2; and *r*_1_, *r_2_*, and *c* are random numbers between 0 and 1,

(30) $$\vec{D^{\prime }}=\left|\vec{X^{\prime }}\,\left(t\right)-\vec{X}\right|$$

Chaos has the characteristics of randomness, regularity, and ergodicity. When solving the function optimization problem, the diversity of the population can be maintained, and the global search ability can be improved. Tent (Shan et al., 2005)has better ergodic uniformity and can improve the search speed of the algorithm, and it can generate more evenly distributed values between [0,1]. The formula is as follows:

(31) $$z_{i+1}=\left\{ \begin{array}{cc} \frac{z_{i}}{u} & 0\leq z_{i}<0\\ \frac{1-z_{i}}{1-u} & u\leq z_{i}\leq 1 \end{array}\right.$$

When *u* = 1/2, tent is the most classical form. The sequence of this form has uniform distribution and approximately uniform distribution density for different parameters. Therefore, the formula of the tent chaotic map in this paper is

(32) $$z_{i+1}=\left\{ \begin{array}{cc} 2\,z_{i} & 0\leq z_{i}<\frac{1}{2}\\ 2\,\left(1-z_{i}\right) & \frac{1}{2}\leq z_{i}\leq 1 \end{array}\right.$$

The sine cosine algorithm is a new intelligent optimization algorithm proposed by Mirjalili in 2016. It is based on the mathematical model of outward sine and cosine wave or the wave in the direction of the optimal solution. Using multiple random variables and adaptive variables to calculate the current solution location, different regions in the space can be searched, effectively avoiding local optimization and converging to the global optimum.

### ICW Optimization Algorithm

Based on the above algorithm introduction, the ICW optimization algorithm is proposed. To expand the predator–prey of whales, somersault foraging method was added. In this strategy, the position of the candidate solution is regarded as a pivot. Each individual tends to somersault around the pivot to a new position. Therefore, each individual always updates its surrounding location until it finds the best location so far. Because the swarm intelligence algorithm has the disadvantage of falling into local optimization, in order to obtain better global optimization ability and increase the search range, the sine cosine mathematical model is introduced, which makes the optimization direction fluctuate outward or to the direction of the optimal solution. The chaos is added to the sine cosine model to further expand the coverage of the solution space. This makes it easy for the algorithm to escape from the local optimal solution, thus maintaining the diversity of the population and improving the global search ability. In general, this algorithm achieves the desired outcome.

The details of the algorithm are as follows:

Step 1. Introduce the parameters and generate the initial population.
Step 2. Calculate the fitness value of the current population and find the optimal solution, which is the minimum fitness value.
Step 3. Obtain the parameters for every iteration.
Step 4. Generate random number p and determine the relationship between *p* and 0.5. If *p* < 0.5, enter step 5; otherwise, enter step 6.
Step 5. Generate | A| and determine the relationship between | A| and 1. If | A| < 1, select the current optimal location for the update operation. Otherwise, select a random location for the update operation.
Step 6. Generate *c* and determine the relationship between *c* and 0.5. If *c* < 0.5, use the spiral upward mechanism to update the position; otherwise, use the somersault mode to update the position.
Step 7. Use sines and cosines with chaos to increase the global search capability and get the set of desirable populations.
Step 8. Select populations that satisfy the constraint combinations.
Step 9. Increase the number of iterations to determine the relationship between the current iteration number and the maximum iteration number. If the current iteration number is less than the maximum, step 2 is performed; otherwise, step 10 is performed.
Step 10. Calculate the fitness function of the desirable populations and select the optimal populations as the final result.

In this work, the conversion method between numbers and letters is as follows: 0-C,1-T,2-A,3-G.

**FIGURE 3:**
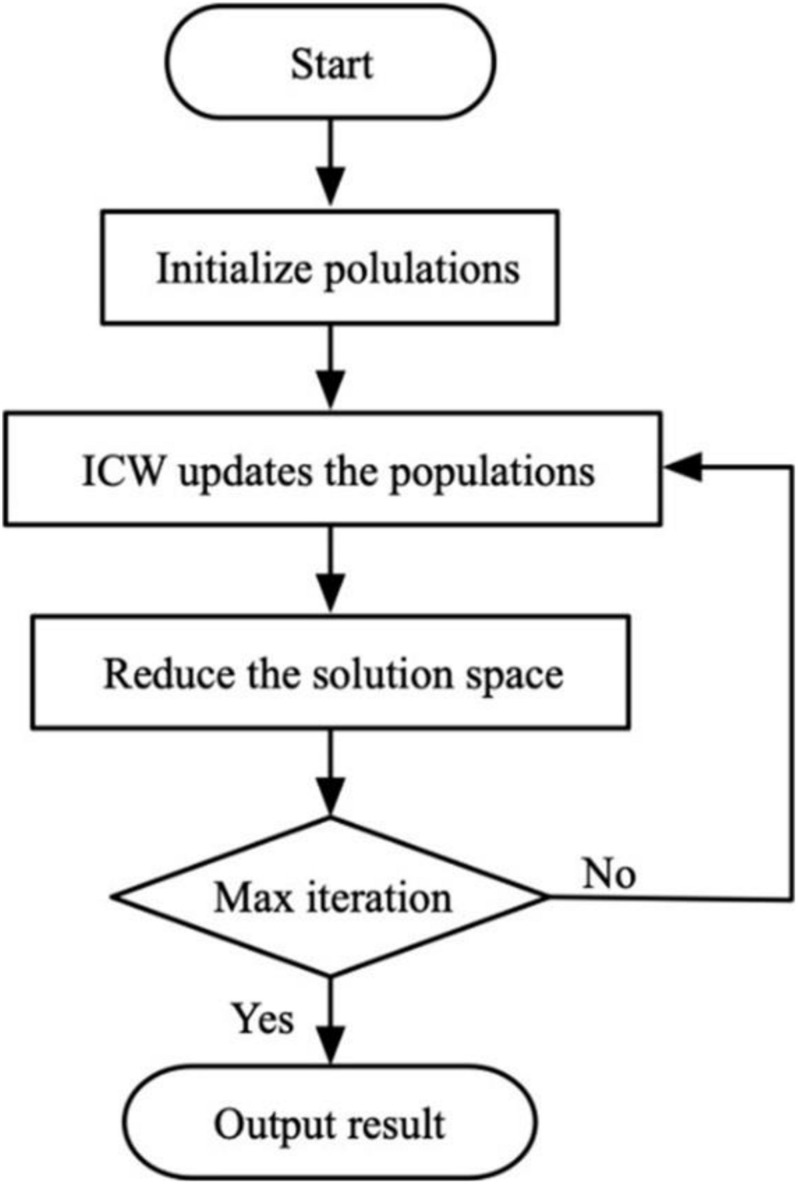
Flow chart of ICW.

**FIGURE 4:**
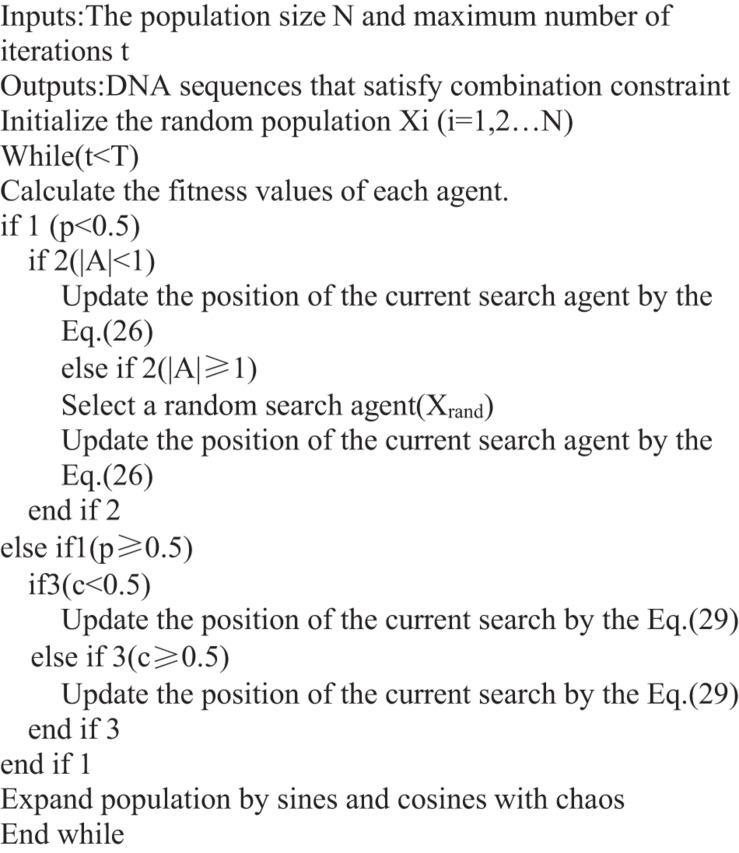
Pseudo-code of the ICW optimization algorithm.

The flow chart of the ICW optimization algorithm is shown in Figure 3, and the Figure 4 presents the pseudo-code of a general implementation.

### Benchmark Test Functions

To better demonstrate the performance of the ICW optimization algorithm, we tested 23 benchmark functions that are widely used. It is worth noting that because each algorithm focuses on solving different types of problems, not every algorithm can get the minimum value of all functions.

For the 23 test functions, the functions can be divided into three categories according to the function types (Digalakis and Margaritis, 2001), namely, unimodal benchmark function (F1–F7), multimodal benchmark function (F8–F13), and fixed-dimension multimodal benchmark function (F14–F23). The equations of the different types of functions are listed in the Supplementary Material. In the table of the Supplementary Material, Function represents benchmark functions; Dim represents function dimension; rang represents the definition domain value of Function; and *f*_*min*_ represents the optimal solution of Function. The unimodal benchmark function has only one global optimal value, so it can be used to test the benchmark development ability of the algorithm. The multimodal benchmark function has one optimal value and several local optimal values. The number of optimal values increases with the increase of the dimension, so it can be used to test the exploration ability of the algorithm and its ability to jump out of the local optimum. Like the multimodal benchmark function, the fixed-dimension multimodal function has only one global optimal value and many local optimal solutions. However, the solution space of the multi-modal function with fixed dimension is very small, so the step size should be adjusted adaptively.

We compared our algorithm with the algorithms HSWOA (Li et al., 2020), WOA (Mirjalili and Lewis, 2016), GA (Heidari et al., 2019), PSO (Yin et al., 2020), FPA (Yang et al., 2014), GWO (Mirjalili et al., 2014), FA (Gandomi et al., 2011), MFO (Mirjalili, 2015), TLBO (Rao et al., 2011), and DE (Yin et al., 2020). We compared the average (AVG) and standard deviation (STD) of 23 benchmark functions. Other conditions remain unchanged, and each function is iterated 500 times and run 30 times. This operation ensures the validity of the test. The test results are shown in Tables 1, 2. Table 1 shows the results of F1–F13 functions calculated by different algorithms, and Table 2 shows the results of the remaining functions.

**TABLE 1:** Result of benchmark functions (F1–F13) with 30 dimensions.

ID	Metric	ICW	HSWOA	WOA	GA	PSO	FPA	GWO	FA	MFO	TLBO	DE
F1	AVG	**0.00E+00**	2.71E−91	1.41E-03	1.03E+03	1.83E+04	2.01E+03	1.18E−27	7.11E−03	1.01E+03	2.17E−89	1.33E−03
	STD	0.00E+00	1.24E−90	4.91E-30	5.79E+02	3.01E+03	5.60E+02	1.47E−27	3.21E−03	3.05E+03	3.14E−89	5.92E−04
F2	AVG	**0.00E+00**	7.03E−58	1.06E-21	2.47E+01	3.58E+02	3.22E+01	9.71E−17	4.34E−01	3.19E+01	2.77E−45	6.83E−03
	STD	0.00E+00	2.94E−57	2.93E-21	5.68E+00	1.35E+03	5.55E+00	5.60E−17	1.84E−01	2.06E+01	3.11E−45	2.06E−03
F3	AVG	**0.00E+00**	1.11E+04	5.39E-07	2.65E+04	4.05E+04	1.41E+03	5.12E−05	1.66E+03	2.43E+04	3.91E−18	3.97E+04
	STD	0.00E+00	4.54E+03	2.93E-06	3.44E+03	8.21E+03	5.59E+02	2.03E−04	6.72E+02	1.41E+04	8.04E−18	5.37E+03
F4	AVG	**0.00E+00**	3.22E+01	0.07E+00	5.17E+01	4.39E+01	2.38E+01	1.24E−06	1.11E−01	7.00E+01	1.68E−36	1.15E+01
	STD	0.00E+00	0.07E+01	0.39E+00	1.05E+01	3.64E+00	2.77E+00	1.94E−06	4.75E−02	7.06E+00	1.47E−36	2.37E+00
F5	AVG	**2.54E+01**	2.76E+01	2.78E+01	1.95E+04	1.96E+07	3.17E+05	2.70E+01	7.97E+01	7.35E+03	2.54E+01	1.06E+02
	STD	0.25E0+00	0.58E+00	0.76E+00	1.31E+04	6.25E+06	1.75E+05	7.78E−01	7.39E+01	2.26E+04	4.26E−01	1.01E+02
F6	AVG	0.11E+00	0.33E+00	3.11E+00	9.01E+02	1.87E+04	1.70E+03	8.44E−01	6.94E−03	2.68E+03	3.29E−05	1.44E−03
	STD	0.03E+00	0.18E+04	0.53E+00	2.84E+02	2.92E+03	3.13E+02	3.18E−01	3.61E−03	5.84E+03	8.65E−05	5.38E−04
F7	AVG	**6.19E-05**	1.29E−03	1.42E-03	1.91E−01	1.07E+01	3.41E−01	1.70E−03	6.62E−02	4.50E+00	1.16E−03	5.24E−02
	STD	6.37E-05	1.20E−03	1.14E−03	1.50E−01	3.05E+00	1.10E−01	1.06E−03	4.23E−02	9.21E+00	3.63E−04	1.37E−02
F8	AVG	*−*1.23E+04	−1.11E+04	−5.08E+03	−**1.26E+04**	−3.86E+03	−6.45E+03	−5.97E+03	−5.85E+03	−8.48E+03	−7.76E+03	−6.82E+03
	STD	5.26E+02	1.50E+03	6.95E+02	4.51E+00	2.49E+02	3.03E+02	7.10E+02	1.16E+03	7.98E+02	1.04E+03	3.94E+02
F9	AVG	**0.00E+00**	9.01E+00	0.00E+00	9.04E+00	2.87E+02	1.82E+02	2.19E+00	3.82E+01	1.59E+02	1.40E+01	1.58E+02
	STD	0.00E+00	1.44E+01	0.00E+00	4.58E+00	1.95E+01	1.24E+01	3.69E+00	1.12E+01	3.21E+01	5.45E+00	1.17E+01
F10	AVG	**8.88E-16**	2.78E−15	7.40E+00	1.36E+01	1.75E+01	7.14E+00	1.03E−13	4.58E−02	1.74E+01	6.45E−15	1.21E−02
	STD	0.00E+00	1.80E−15	9.89E+00	1.51E+00	3.67E−01	1.08E+00	1.70E−14	1.20E−02	4.95E+00	1.79E−15	3.30E−03
F11	AVG	**0.00E+00**	**0.00E+00**	2.89E-04	1.01E+01	1.70E+02	1.73E+01	4.76E−03	4.23E−03	3.10E+01	**0.00E+00**	3.52E−02
	STD	0.00E+00	0.00E+00	1.58E+03	2.43E+00	3.17E+01	3.63E+00	8.57E−03	1.29E−03	5.94E+01	0.00E+00	7.20E−02
F12	AVG	5.06E-03	6.90E−00	3.39E-01	4.77E+00	1.51E+07	3.05E+02	4.83E−02	3.13E−04	2.46E+02	**7.35E**−**06**	2.25E−03
	STD	2.54E-03	7.06E−00	0.21E+00	1.56E+00	9.88E+06	1.04E+03	2.12E−02	1.76E−04	1.21E+03	7.45E−06	1.70E−03
F13	AVG	0.10E-00	4.52E−00	1.88E+00	1.52E+01	5.73E+07	9.59E+04	5.96E−01	**2.08E**−**03**	2.73E+07	7.89E−02	9.12E−03
	STD	9.40E-02	9.14E−00	3.66E+01	4.52E+00	2.68E+07	1.46E+05	2.23E−01	9.62E−04	1.04E+08	8.78E−02	1.16E−02

*Bold values represent the optimal value of the function in the table.*

**TABLE 2:** Results of benchmark functions (F14–F23) with 30 dimensions.

ID	Metric	ICW	HSWOA	WOA	GA	PSO	FPA	GWO	FA	MFO	TLBO	DE
F14	AVG	3.73E-00	2.18E−00	2.11E+00	**9.98E**−**01**	1.39E+00	9.98E−01	4.17E+00	3.51E+00	2.74E+00	**9.98E**−**01**	1.23E+00
	STD	4.17E-00	2.11E−00	2.49E+00	4.52E−16	4.60E−01	2.00E−04	3.61E+00	2.16E+00	1.82E+00	4.52E−16	9.23E−01
F15	AVG	**3.99E-04**	5.25E−04	5.72E−04	3.33E−02	1.61E−03	6.88E−04	6.24E−03	1.01E−03	2.35E−03	1.03E−03	5.63E−04
	STD	1.30E-04	2.54E−04	3.24E−04	2.70E−02	4.60E−04	1.55E−04	1.25E−02	4.01E−04	4.92E−03	3.66E−03	2.81E−04
F16	AVG	*−***1.03E+00**	−1.03E+00	−**1.03E**−**00**	−3.78E−01	−**1.03E+00**	−**1.03E+00**	−**1.03E+00**	−**1.03E+00**	−**1.03E+00**	−**1.03E+00**	−**1.03E+00**
	STD	1.94E-10	1.32E−08	4.20E−07	3.42E−01	2.95E−03	6.78E−16	6.78E−16	6.78E−16	6.78E−16	6.78E−16	6.78E−16
F17	AVG	**3.98E-01**	3.97E−01	3.97E−01	5.24E−01	4.00E−01	**3.98E**−**01**	**3.98E**−**01**	**3.98E**−**01**	**3.98E**−**01**	**3.98E**−**01**	**3.98E**−**01**
	STD	8.20E-07	1.19E−07	2.70E−05	6.06E−02	1.39E−03	1.69E−16	1.69E−16	1.69E−16	1.69E−16	1.69E−16	1.69E−16
F18	AVG	**3.00E+00**	**3.00E+00**	**3.00E+00**	**3.00E+00**	3.10E+00	**3.00E+00**	**3.00E+00**	**3.00E+00**	**3.00E+00**	**3.00E+00**	**3.00E+00**
	STD	1.65E-14	5.98E-07	4.22E-15	0.00E+00	7.60E−02	0.00E+00	4.07E−05	0.00E+00	0.00E+00	0.00E+00	0.00E+00
F19	AVG	*−***3.86E+00**	−**3.86E+00**	−3.85E+00	−3.42E+00	−**3.86E+00**	−**3.86E+00**	−**3.86E+00**	−**3.86E+00**	−**3.86E+00**	−**3.86E+00**	−**3.86E+00**
	STD	6.85E-06	3.19E−03	2.70E−03	3.03E−01	1.24E−03	3.16E−15	3.14E−03	3.16E−15	1.44E−03	3.16E−15	3.16E−15
F20	AVG	*−*3.2821	−3.27	−2.98105	−1.61351	−3.11088	−**3.2951**	−3.25866	−3.28105	−3.23509	−3.24362	−3.27048
	STD	0.057049	0.060296	0.376653	0.46049	0.029126	0.019514	0.064305	0.063635	0.064223	0.15125	0.058919
F21	AVG	*−***10.142**	−8.7129	−7.04918	−6.66177	−4.14764	−5.21514	−8.64121	−7.67362	−6.8859	−8.64525	−9.64796
	STD	0.011404	2.4655	3.629551	3.732521	0.919578	0.008154	2.563356	3.50697	3.18186	1.76521	1.51572
F22	AVG	*−***10.3907**	−8.1948	−8.18178	−5.58399	−6.01045	−5.34373	−10.4014	−9.63827	−8.26492	−10.2251	−9.74807
	STD	0.015752	2.9941	3.29202	2.605837	1.962628	0.053685	0.000678	2.293901	3.076809	0.007265	1.987703
F23	AVG	*−*10.527	−8.7416	−9.34238	−4.69882	−4.72192	−5.29437	−10.0836	−9.75489	−7.65923	−10.0752	−**10.5364**
	STD	0.011063	2.8113	2.414737	3.256702	1.742618	0.356377	1.721889	2.345487	3.576927	1.696222	8.88E−15

*Bold values represent the optimal value of the function in the table.*

As shown in Tables 1, 2, the ICW optimization algorithm is better than the other algorithms in most function values. In particular, our algorithm achieves the optimal values for unimodal functions F1–F4, F9, F11 and F16, F18, F19 of the multimodal functions. In addition, in the functions F5, F7, F10, F11, F15–F17, F19, F21, and F22, the improved algorithm is significantly better than the other algorithms in the table. With respect to the original algorithm WOA, by comparing the average value of the two algorithms, it can be found that the improved ICW optimization algorithm significantly enhances the development ability and gives results that are closer to the global optimization. For standard deviation, the new algorithm has better stability. Compared with the other functions in the table, the ICW optimization algorithm occupies nearly 75% of the optimal value. So, this algorithm has a strong competitive advantage.

**FIGURE 5:**
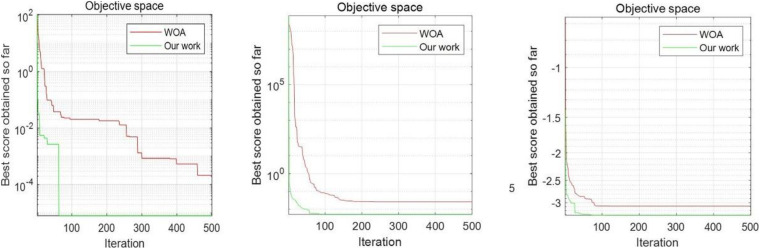
Test functions of functions F7, F12, and F20.

To further illustrate that the improved algorithm has more advantages than the WOA algorithm, the single peak test function, multiple test function, and fixed image of the multimodal function are drawn. We do not select the function that reaches the optimal value, which ensures universal optimization of the new algorithm and enhances the persuasiveness. As shown in the figure, F7, F12, and F20 are selected Figure 5. The unimodal benchmark function F7 can jump out of the local optimal solution and converge to the global optimum. The multimodal benchmark function F12 and the fixed-dimension multimodal benchmark function F20 can converge to the local optimal solution quickly.

## Results and Analysis

In this article, the results are obtained by running the algorithm in MATLAB R2018a. The computer had the Windows 10 operating system, Intel (R) CPU2.70 GHz, and ARM 8.00 GB. The minimum stem length and ring length of the hairpin structure were 6. The minimum continuity threshold was set to 2. In the continuous case, the threshold of similarity and H-measure were six, and the threshold of discontinuous similarity and discontinuous H-measure were 0.17. In addition, Tm was obtained by using the proximity model. The concentration of DNA was set at 10 nM, and salt concentration was set at 1 M.

This part compares ICW with other algorithms, namely NACST/Seq (Shin et al., 2005), DEPT (Chaves-González and Vega-Rodríguez, 2014), MO-ABC (Chaves-González et al., 2013), pMO-ABC (Chaves-González and Martínez-Gil, 2019), and HSWOA (Li et al., 2020). We compare the average values of the above algorithms for continuity, hairpin structure, H-measure, similarity, and melting temperature. We also input the optimal sequence of the algorithm into NUPACK for experimental simulation and compared the simulation results.

**FIGURE 6:**
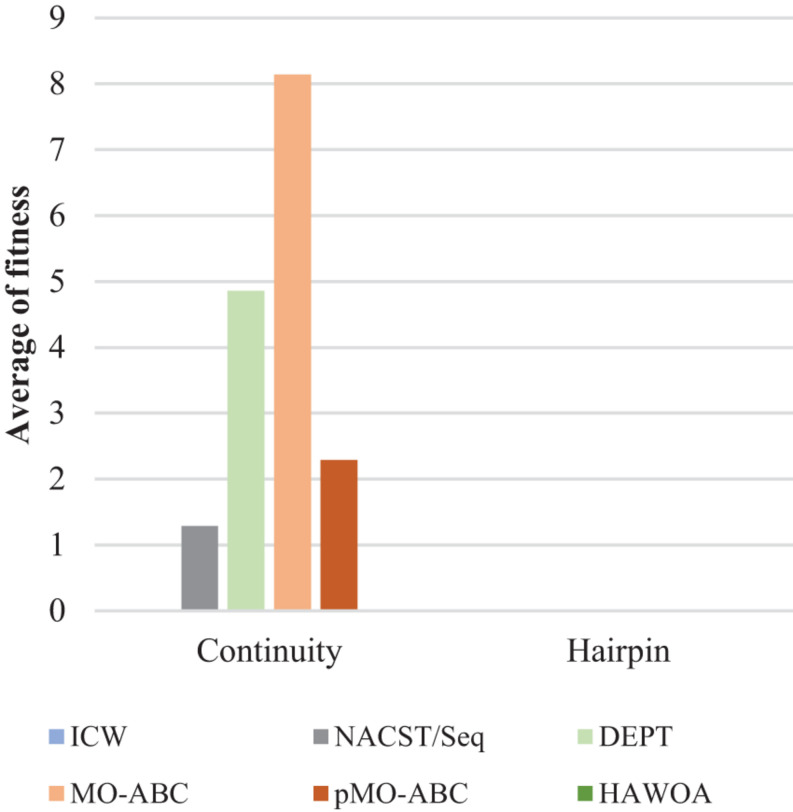
Average fitness of continuity and hairpin for ICW and other algorithms.

**FIGURE 7:**
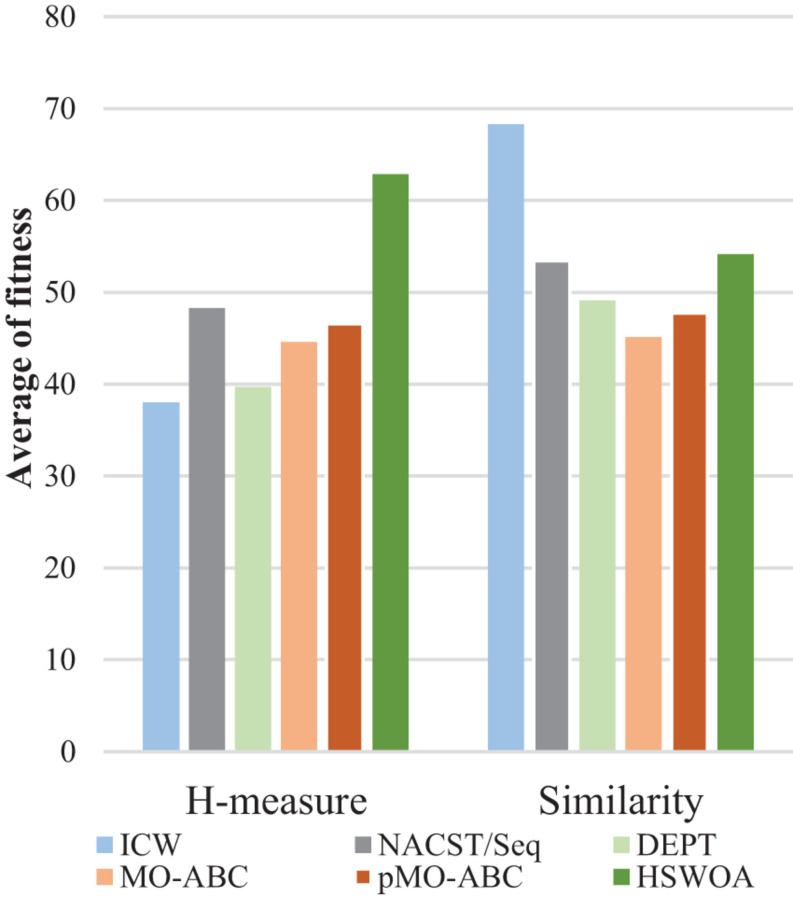
Average fitness of H-measure and similarity for ICW and other algorithms.

**FIGURE 8:**
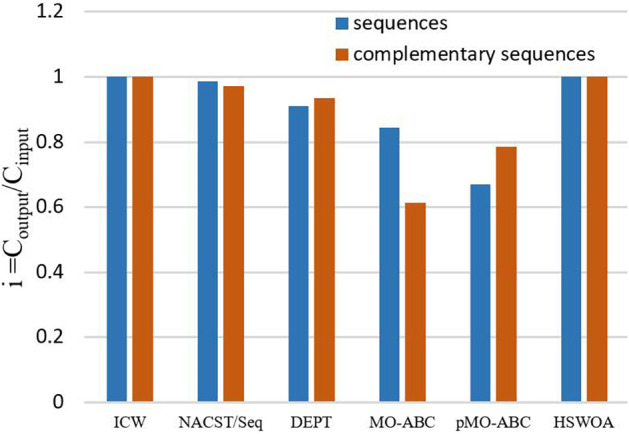
Value of i for different algorithms.

**FIGURE 9:**
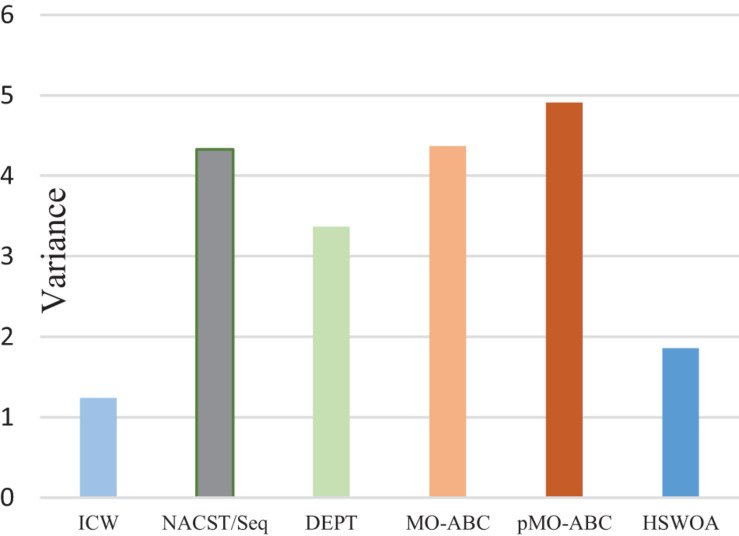
Variance of different algorithms.

Table 3 compares the sequences obtained by our proposed algorithm with those of other algorithms. Seven sequences were used in Table 3; each sequence has 20 bases. According to the average values of continuity, hairpin structure, H-measure, similarity, and Tm in Table 3, Figures 6–9, and Table 4, the results show that our work outperforms other algorithms in terms of continuity and hairpin structure. In the aspect of H-measure, the results obtained by our algorithm are much better than other algorithms, which indicates that our algorithm can effectively avoid non-specific hybridization. In the reaction solution, the DNA sequence can also maintain the maximum value. In general, it can effectively avoid non-specific hybridization between sequences. The GC content is always maintained at 50%, representing that the sequences obtained by our algorithm have stable thermodynamic properties.

**TABLE 3:** Comparing sequences from NACS/Seq, DEPT, MO-ABC, pMO-ABC, and HSWOA.

Seq.	C	P	H	S	Tm	GC%	Seq.	C	P	H	S	Tm	GC%
**Our work**							**NACST/Seq [21]**						
CCTCTCCATCCTTATCCTTC	0	0	35	66	60.88	50	CTCTCATCTCTCCGTTCTTC	0	0	37	58	61.43	50
CCAGACCAATACAGAACCAC	0	0	50	57	62.54	50	TATCCTGTGGTGTCCTTCCT	0	0	45	57	64.46	50
CTCCTCTTCTCCTTCTTCTC	0	0	28	82	60.72	50	GTATTCCAAGCGTCCGTGTT	0	0	55	49	65.29	50
CACAACCAATCACTCTCACC	0	0	38	65	63.10	50	TCTCTTACGTTGGTTGGCTG	0	0	51	53	64.63	50
CCACCTGACCGACTAATAAC	0	0	48	61	62.02	50	CTCTTCATCCACCTCTTCTC	0	0	43	58	61.38	50
CCAACCACTCTTCTACAACC	0	0	37	72	62.47	50	ATTCTGTTCCGTTGCGTGTC	0	0	52	56	65.82	50
CCTTCTTCTCTCTCTCTCTC	0	0	30	75	60.13	50	AAACCTCCACCAACACACCA	9	0	55	43	66.71	50
**DEPT [23]**							**MO-ABC [22]**						
CCATTCCTTAACCTCTCTCC	0	0	59	39	61.39	50	GTAAGGAAGGCAAGGCAGAA	0	0	42	54	64.70	50
ACACACACACACACACACAC	0	0	27	49	65.85	50	GTTGGTGGTTGTTGGTGGTT	0	0	46	36	66.00	50
GGAAGGAGGAGGAAGAAGAA	0	0	37	45	62.83	50	GGAGACGGAATGGAAGAGTA	0	0	44	55	62.93	50
GAGAGAAGAGAAGAGGCCAA	0	0	39	53	63.17	50	CCATTCTTCTCTTCTCTCCC	9	0	67	22	61.39	50
ACCACAACAACAACACACCC	9	0	29	55	65.96	50	AGGAGAGGAGAGGAGGAAAA	16	0	31	53	63.80	50
GGAGCAATGGAGAATAAGGG	9	0	48	47	62.42	50	ATAAGAGAGAGAGAGAGGGG	16	0	34	51	61.11	50
CCATACCAGCCAACCGAAAA	16	0	42	56	65.33	50	GAGCCAACAGCCAACCAAAA	16	0	48	45	66.40	50
**pMO-ABC [28]**							**HSWOA [36]**						
GGTGGTATTGGTGGTATTGG	0	0	47	47	62.64	50	CTCGTCTAACCTTCTTCAGC	0	0	63	51	62.28	50
CTTCTCTTCTCTTGCCGCTT	0	0	39	56	64.70	50	CTGTGTGGAATGCAAGGATG	0	0	64	48	63.82	50
CTCTCTCTCTCACTCTCTCA	0	0	41	48	61.32	50	CGAGCGTAGTGTAGTCATCA	0	0	63	69	63.56	50
AACAACCTCCACACCGAACA	0	0	62	32	66.69	50	AGTTACAGGACACCACCGAT	0	0	65	51	66.39	50
TGTGGTTGGTTAGTCGGTTG	0	0	46	49	63.80	50	CAGTAGCAGTCATAACGAGC	0	0	64	56	62.69	50
TGGTGTTGCTGGTGTAGGTT	0	0	48	51	66.46	50	GCATAGCACATCGTAGCGTA	0	0	59	54	64.60	50
CTCTCATTCCTTCTTACCCC	16	0	43	51	61.40	50	TGGACCTTGAGAGTGGAGAT	0	0	62	50	64.44	50

**TABLE 4:** Comparing the melting temperatures of the various algorithm sequences.

	ICW	NACST/Seq	DEPT	MO-ABC	pMO-ABC	HSWOA
Var	1.24	4.33	3.37	4.37	4.91	1.86

In Figure 6, the result shows the average fitness of continuity and hairpin for different algorithms. From the bar graph, it can be clearly seen that the sequence obtained by the ICW optimization algorithm was better than that obtained by other algorithms in terms of continuity and hairpin structure. ICW optimization algorithm obtained the minimum value. Therefore, the sequence obtained by our algorithm can effectively avoid the generation of secondary structures.

Using Table 3, we calculated the average fitness of different algorithms for H-measure and similarity (Figure 7). It can be clearly seen from the graph that our algorithm obtains the minimum H-measure. However, our algorithm is different from other comparable algorithms in terms of the similarity.

After comparing the above average fitness values, we also studied the evaluation results in NUPACK. Most DNA solution experiments are performed at room temperature, so the temperature was set at 25°C. We input the seven sequences (optimal sequences of each algorithm) of different algorithms in Table 3 into NUPACK. After evaluation, the values of *C*_*input*_ and *C*_*output*_ were calculated, and the value of *i* was obtained, as shown in Figure 8.

Figure 8 shows the values of *i* for different algorithms. It should be noted that the closer *i* is to 1, the higher is the quality of the sequences. In the figure, each algorithm has two columnar regions. The first represents seven sequences of the algorithms in Table 3, and the second column region represents the complement sequence of these seven sequences. First, consider the sequences in Table 3. In the histogram, ICW and HSWOA, which is our previous work, both have the value 1 for *i*. In the HSWOA algorithm, the triplet-bases unpaired constraint is added to form a constraint combination to control a single sequence so that the sequence does not react with itself. In the ICW optimization algorithm, PSC is added to form a new constraint combination to reduce the reaction between different sequences. From the value of *i*, the new algorithm with constraint is better performing than other algorithms.

In addition, considering the double stranded structure of DNA, the complements of seven DNA sequences were evaluated. The evaluation results are shown in Figure 8. In the figure, the values of the ICW optimization algorithm and HSWOA algorithm are 1, indicating that there is no reaction between complementary sequences, which is what we expected. In addition, seven DNA sequences and complements were input into NUPACK for evaluation, finding the Fourteen sequences were complete reactions, which were complementary to each other.

Table 4 and Figure 9 show the variance of the melting temperature of different algorithms. The values in the table are calculated from the Tm values in Table 4. In DNA computing, to ensure the consistency of the biochemical reactions of DNA molecules, all the DNA molecules participating in the biochemical reactions should be uniform. In other words, if the variance is small, the melting temperature change will be small, and the probability of achieving the desired result will be increased.

The significance of this work is evaluated again by the variance of melting temperature (Tm) and the value of *i.* In order to ensure the consistency of the work, in the comparison of indicators, the control group still chooses sequence a and sequence b and compares with the average value of the indicators in this work. The variance of the Tm of the DNA sequence obtained by the pMO-ABC algorithm is 1.65, while the variance of the Tm in this work is 1.24, which is reduced by 33.1%. Smaller variance of Tm is more conducive to controlling the temperature during the reaction, and smaller temperature fluctuation is more conducive to the reaction. The *i* value of the two sequences of *a* and *b* is *i* = 0.285, while the *i* of the set of DNA sequences in this work is all 1. The closer i is to 1, the higher the sequence quality.

**FIGURE 10:**
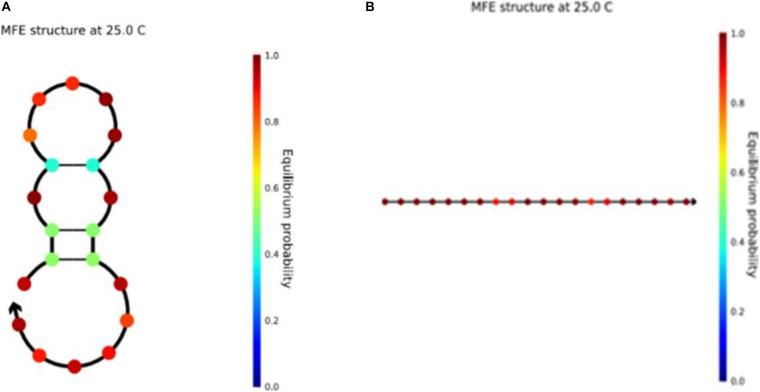
**(A)** The structure of sequence ACAGTCGTTAAATGGGAGTC in NUPACK. **(B)** The structure of sequence CCTCTCCATCCTTATCCTTC in NUPACK.

It is worth noting that the sequence structure of the unstable available structure in the previous article (Li et al., 2020) is shown in Figure 10A, and this structure has also been changed in this work. Seven optimized sequences satisfying the new combinatorial constraints were input into NUPACK at the same time. We found that all the sequences were linear structures. They were stable and available structures that could improve the accuracy of DNA calculation. As shown in the figure, the color bar on the right shows the stability of the sequence. The closer the color is to red, the more stable it is. Compared with the two graphs, the sequence structure of Figure 10B is more stable. In the graph of the two DNA sequences, the figures represent the stable structure of the sequences. In addition, other DNA sequence structures obtained from our work are presented in the Supplementary Material.

## Conclusion

In this study, we input existing DNA sequences into NUPACK and evaluated them. It was found that the DNA sequences may react with each other owing to base complementary pairing. Therefore, we propose a new constraint, PSC, to solve this problem. In addition, due to the double strand structure of DNA, if the A-T base is located at one end of the DNA sequence and its complementary sequence, there may be a gap, which leads to a decrease of the accuracy of calculation. The proposed Close-ending constraint can effectively avoid the generation of such sequences. These two new constraints were fused into the previous constraint combination to form a new combination constraint. Then, the new ICW optimization algorithm was used to obtain the sequences satisfying the new combination constraints, and the sequence results were analyzed. The analysis results show that the current minimum is obtained on continuity, hairpin, and H-measure. This shows that the sequence greatly improves the ability to avoid secondary structures. In terms of the Tm value, the minimum variance was obtained by calculation, which ensured that the DNA molecules participating in biochemical reactions were more uniform and improved the thermodynamic stability. When the sequences were input into NUPACK for evaluation, the concentration of the obtained sequence in the solution was the same as before, indicating that the DNA sequence did not react with itself or other sequences in the solution, and the DNA sequence was stable and available in the solution, which improved the accuracy of DNA calculation.

In the future, to produce stable DNA sequence and ensure the accuracy of DNA computing, we will take the optimization of the DNA sequence set as the primary task. With respect to the algorithm, we will further optimize it. At the same time, reducing the similarity of DNA sequence sets and exploring the linear structure of DNA sequences in solution will also be two important aspects. In addition, to expand the application of DNA computing, we will also work with the machine learning (Song et al., 2019; Gong et al., 2020) and bioinformatics (Zou et al., 2020).

## Data Availability Statement

The raw data supporting the conclusions of this article will be made available by the authors, without undue reservation.

## Author Contributions

XL and ZW designed the DNA sequences, analyzed the data and the performance, and wrote the manuscript. BW and TS supervised the work, evaluated the performance, and revised the manuscript. All authors have read and agreed to the published version of the manuscript.

## Conflict of Interest

The authors declare that the research was conducted in the absence of any commercial or financial relationships that could be construed as a potential conflict of interest.
