# Supplementary material for: Stable DNA Sequence Over Close-Ending and Pairing Sequences Constraint
Source: Front Genet. 2021 May 17;12:644484. doi: 10.3389/fgene.2021.644484 (PMC8165483; doi:10.3389/fgene.2021.644484)
Supplement: Supplementary file 1 [file Data_Sheet_1.docx]

Supplementary Material

This document is a supplement to the text of article Stable DNA sequence over Close-ending and Pairing Sequences Constraint. The value of M mentioned in formula (24) is verified in the first part of this document. The benchmark Test Functions mentioned in section 3.3 give details in the second part of this file. In the result analysis section, details of other work sequences mentioned in the text are given in the third part of this document. The following is the main content.

# The Value of M


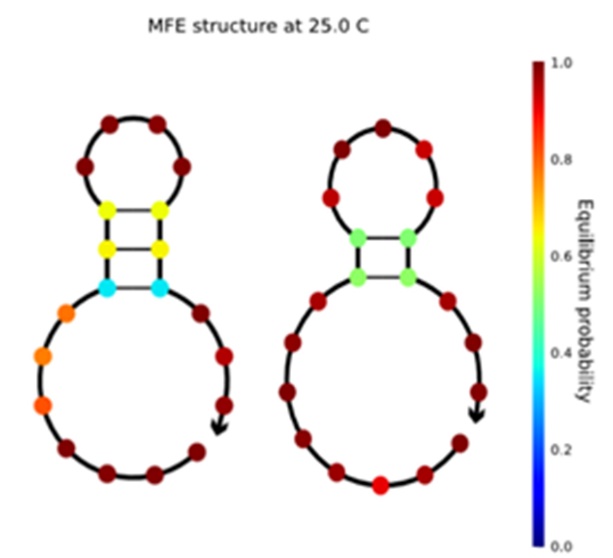


FIG.1B The respective structures of sequences a and c.

In this section, we introduced the value of the text formula (24). Still take Fig. 2 of the text as an example to operate sequence a. What needs to be explained is sequence a: AACAACCTCCACACCG AACA, and sequence b: TGGTGTTGCTGGTGTAGGTT. Among them, the crossed parts were the base participating in complementary pairing, and its stability diagram is shown in the FIG.1A. It should be noted that i = 0.5 of the structure in the solution. Under the condition that the GC content is 50%, the first lineation is changed to ATCCT, the second is changed to ACACGG, and then the two DNA sequences do not react with each other in the solution, and i = 1. And setting c as the changed sequence, sequence c: AACATCCTCC ACACGGAACA. We show the structure of the two sequences in the FIG.1B.


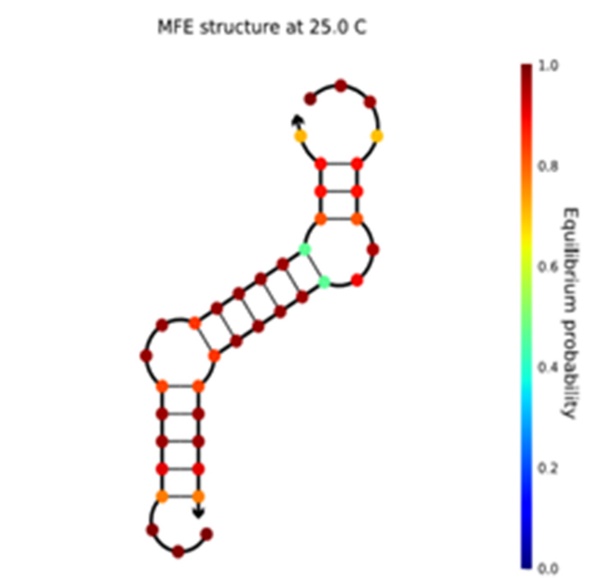


FIG.1A The structure generated by sequences a and b.

As shown in the figure above, for two sequences, when the number of paired bases is controlled at 4, the situation and probability of interaction between sequences will be greatly reduced. Therefore, the value of M = 4 is conducive to the accurate hybridization reaction of DNA sequence.

# Details of 23 Benchmark Function

**Table S1**. Description of Unimodal benchmark functions

| Function | Dim | Range | f_min_ |
| --- | --- | --- | --- |
| $\text{F}_{1}(x)=\sum_{i=1}^{n} X_{i}^{2}$ | 50 | [-100,100] | 0 |
|  | 50 | [-10,10] | 0 |
| $\text{F}_{3}(x)=\sum_{i=1}^{n} (\sum_{j-1}^{i} x_{j})^{2}$ | 50 | [-100,100] | 0 |
| $F_{4}(x)=\max_{i}\text{\{}\vert x_{i}\vert,1\leq i\leq n\}$ | 50 | [-100,100] | 0 |
| $\text{F}_{5}(x)=\sum_{i=1}^{n-1} [100(x_{i+1}-x_{i}^{2})^{2}+(x_{i}-1)^{2}]$ | 50 | [-30,30] | 0 |
| $\text{F}_{6}(x)=\sum_{i=1}^{n} ([x_{i}+0.5])^{2}$ | 50 | [-100,100] | 0 |
| $F_{7}(x)=\sum_{i=1}^{n} ix_{i}^{4}+random[0,1)$ | 50 | [-1.28,1.28] | 0 |

**Table S2**. Description of Multimodal benchmark functions.

| Function | Dim | Range | f_min_ |
| --- | --- | --- | --- |
| $F_{8}(x)=\sum_{i=1}^{n} -x_{i}\sin(\sqrt{\vert x_{i}\vert})$ | 50 | [-500,500] | -418.9829*5 |
| $F_{9}(x)=\sum_{i=1}^{n} [x_{i}^{2}-10\cos(2\pi x_{i})+10]$ | 50 | [-5.12,5.12] | 0 |
| $\begin{matrix} F_{10}(x)=-20\exp(-0.2\sqrt{\begin{matrix} \frac{1}{n} & \sum_{i=1}^{n} x_{i}^{2} \end{matrix}})- \\ \text{ }\exp\left(\begin{matrix} \frac{1}{n} & \sum_{i=1}^{n} \cos(2\pi x_{i}) \end{matrix}\right)+20+e \end{matrix}$ | 50 | [-32,32] | 0 |
| $F_{11}(x)=\frac{1}{4000}\sum_{i=1}^{n} x_{i}^{2}-\prod_{i=1}^{n} x_{i}^{2}\cos\left(\frac{x_{i}}{\sqrt{i}}\right)+1$ | 50 | [-600,600] | 0 |
| $\begin{matrix} F_{12}(x)=\frac{\pi}{n}\left\{10\sin(\pi y_{1})+\sum_{i=1}^{n-1} (y_{i}-1)^{2}[1+10\sin^{2}(\pi y_{i+1})+(y_{n}-1)^{2}]\right\} \\ \text{ +}\sum_{i=1}^{n} u(x_{i},10,100,4) \\ y_{i}=1+\frac{x_{i}+1}{4} \\ u(x_{i},a,k,m)=\left\{\begin{matrix} k(x_{i}-a)^{m}\begin{matrix} & x_{i}>a \end{matrix} \\ 0\begin{matrix} & -a<x_{i}<a \end{matrix} \\ k(-x_{i}-a)^{m}\begin{matrix} & x_{i}<-a \end{matrix} \end{matrix}\right. \end{matrix}$ | 50 | [-50,50] | 0 |
| $F_{13}(x)=0.1\left\{\sin^{2}(3\pi x_{1})+\sum_{i=1}^{n} \begin{matrix} (x_{i}-1)^{2}[1+\sin^{2}(3\pi x_{i}+1)]+ \\ (x_{n}-1)^{2}[1+\sin^{2}(2\pi x_{n})] \end{matrix}\right\}$ | 50 | [-50,50] | 0 |
|  |  |  |  |

Table S3. Description of Fixed-dimension Multi-modal benchmark functions.

| Function | Dim | Range | f_min_ |
| --- | --- | --- | --- |
| $F_{14}(x)=(\frac{1}{500}+\sum_{j=1}^{25} \frac{1}{j+\sum_{i=1}^{2} (x_{i}-a_{ij})})^{-1}$ | 2 | [-65,65] | 1 |
| $F_{15}(x)=\sum_{i=1}^{11} [a_{i}-\frac{x_{1}(b_{i}^{2}+b_{i}x_{2})}{b_{i}^{2}+b_{i}x_{3}+x_{4}}]^{2}$ | 4 | [-5,5] | 0.00030 |
| $F_{16}(x)=4x_{1}^{2}-2.1x_{1}^{4}+\frac{1}{3}x_{1}^{6}+x_{1}x_{2}-4x_{2}^{2}+4x_{2}^{4}$ | 2 | [-5,5] | -1.0316 |
| $F_{17}(x)=\left(x_{2}-\frac{5.1}{4\pi^{2}}x_{1}^{2}+\frac{5}{\pi}x_{1}-6\right)^{2}+10\left(1-\frac{1}{8\pi}\right)\cos x_{1}+10$ | 2 | [-5,5] | 0.398 |
| $\begin{matrix} F_{18}(x)=\left[1+\left(x_{1}+x_{2}+1\right)^{2}\left(19-14x_{1}+3x_{1}^{2}-14x_{2}+6x_{1}x_{2}+3x_{2}^{2}\right)\right] \\ \times\left[30+\left(2x_{1}-3x_{2}\right)^{2}\times\left(18-32x_{1}+12x_{1}^{2}+48x_{2}-36x_{1}x_{2}+27x_{2}^{2}\right)\right] \end{matrix}$ | 2 | [-2,2] | 3 |
| $F_{19}(x)=-\sum_{i=1}^{4} c_{i}\exp\left(-\sum_{j=1}^{3} a_{ij}\left(x_{j}-p_{ij}\right)^{2}\right)$ | 3 | [1,3] | -3.86 |
| $F_{20}(x)=-\sum_{i=1}^{4} c_{i}\exp\left(-\sum_{j=1}^{6} a_{ij}\left(x_{j}-p_{ij}\right)^{2}\right)$ | 6 | [0,1] | -3.32 |
| $F_{21}(x)=-\sum_{i=1}^{5} \left[\left(X-a_{i}\right)\left(X-a_{i}\right)^{T}+c_{i}\right]^{-1}$ | 4 | [0,10] | -10.1532 |
| $F_{22}(x)=-\sum_{i=1}^{7} \left[\left(X-a_{i}\right)\left(X-a_{i}\right)^{T}+c_{i}\right]^{-1}$ | 4 | [0,10] | -10.4028 |
| $F_{23}(x)=-\sum_{i=1}^{10} \left[\left(X-a_{i}\right)\left(X-a_{i}\right)^{T}+c_{i}\right]^{-1}$ | 4 | [0,10] | -10.5363 |

Tables S1, S2, and S3 show the reference functions mentioned in the text. Table S1 shows Unimodal benchmark functions, Table S2 shows Multimodal benchmark functions, and Table S3 lists fixed-dimension multi-modal benchmark functions. Where Dim represents the dimension of the function, Range represents the value of the function's domain, and fmin represents the optimal solution of the function. In the test run results, the closer fmin, the more advantages of the algorithm can be reflected.

# Sequences and NUPACK evaluation

FIG.2-7 show the information of the remaining six sequences in the ICW algorithm. As shown in the figure, the color bar on the right side indicates the stability of the current structure, and the color close to red indicates that the stability of the sequence is high. In the six graphs, we can clearly see that the sequence presents a stable linear structure, which is called the stable available structure.


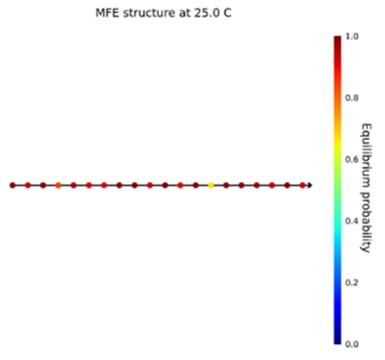

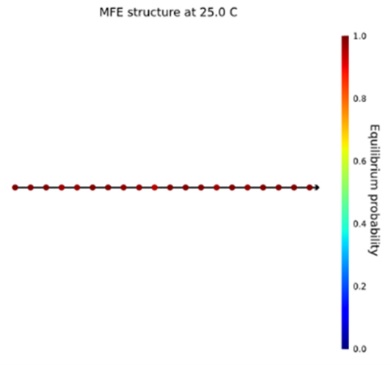

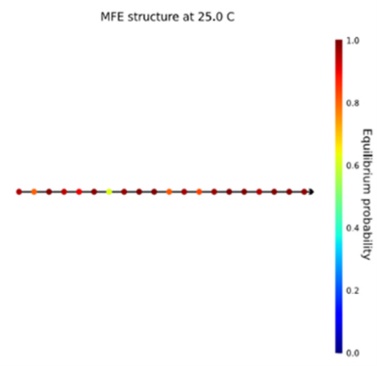


FIG.2 CCAGACCAATACAGAACCAC FIG.3 CTCCTCTTCTCCTTCTTCTC FIG.4 CACAACCAATCACTCTCACC


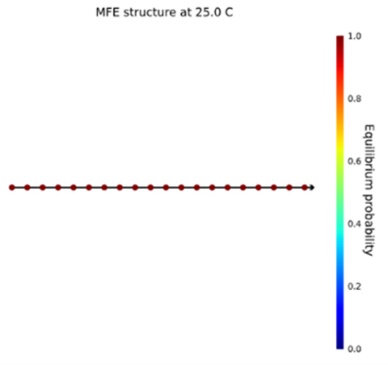

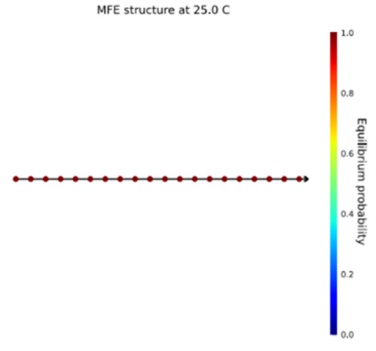

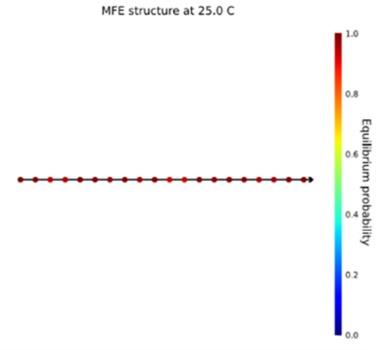


FIG.5 CCACCTGACCGACTAATAAC FIG.6 CCAACCACTCTTCTACAACC FIG.7 CCTTCTTCTCTCTCTCTCTC
